# Competition between Granule Bound Starch Synthase and Starch Branching Enzyme in Starch Biosynthesis

**DOI:** 10.1186/s12284-019-0353-3

**Published:** 2019-12-23

**Authors:** Huaxin Han, Chuantian Yang, Jihui Zhu, Lixia Zhang, Yeming Bai, Enpeng Li, Robert G. Gilbert

**Affiliations:** 1 Jiangsu Key Laboratory of Crop Genetics and Physiology, Key Laboratory of Plant Functional Genomics of the Ministry of Education, Jiangsu Key Laboratory of Crop Genetics and Physiology, College of Agriculture, Yangzhou University, Yangzhou, 225009 People’s Republic of China; 2 Co-Innovation Center for Modern Production Technology of Grain Crops, Yangzhou University, Yangzhou, 225009 China; 3 Crop Breeding and Cultivation Research Institute, Shanghai Academy of Agricultural Sciences, Shanghai, 201403 China; 4 Centre for Nutrition & Food Sciences, Queensland Alliance for Agriculture & Food Innovations, The University of Queensland, QLD, Brisbane, 4072 Australia

**Keywords:** Starch, Biosynthesis, Enzyme actions, Rice, Structural characterization

## Abstract

**Background:**

Starch branching enzymes (SBE) and granule-bound starch synthase (GBSS) are two important enzymes for starch biosynthesis. SBE mainly contributes to the formation of side branches, and GBSS mainly contributes for the synthesis of amylose molecules. However, there are still gaps in the understanding of possible interactions between SBE and GBSS.

**Results:**

Nineteen natural rice varieties with amylose contents up to 28% were used. The molecular structure, in the form of the chain-length distribution (CLDs, the distribution of the number of monomer units in each branch) was measured after enzymatic debranching, using fluorophore-assisted carbohydrate electrophoresis for amylopectin and size- exclusion chromatography for amylose. The resulting distributions were fitted to two mathematical models based on the underlying biosynthetic processes, which express the CLDs in terms of parameters reflecting relevant enzyme activities.

**Conclusions:**

Finding statistically valid correlations between the values of these parameters showed that GBSSI and SBEI compete for substrates during rice starch biosynthesis, and synthesis of amylose short chains involves several enzymes including GBSSI, SBE and SSS (soluble starch synthase). Since the amylose CLD is important for a number of functional properties such as digestion rate, this knowledge is potentially useful for developing varieties with improved functional properties.

## Background

Amylose and amylopectin are the two main components of starch, which is a complex branched glucose polymer. Amylose has moderate molecular weight with a small number of long-chain branches, and amylopectin has large molecular weight with a vast number of short-chain branches (Chiaramonte et al. 2012; Tester et al. 2004).

Concerted actions of a series of biosynthetic enzymes with multiple isoforms, mainly starch synthases (SS), starch branching enzymes (SBE), starch debranching enzymes (DBE) and ADP-glucose pyrophosphorylase polypeptide (AGPase), are involved in starch biosynthesis in cereal endosperms (Akihiro et al. 2005; Hennen-Bierwagen et al. 2008). Granule-bound starch synthase I (GBSSI) is the key enzyme for amylose biosynthesis, predominantly elongating the amylose chains; the soluble starch synthases (SSS) are mainly responsible for amylopectin elongation (Ball et al. 1998; Denyer et al. 2001; Wang et al. 2015). Starch branches are formed by starch branching enzymes (SBE) through transferring an oligosaccharide fragment with a non-reducing end and a (1 → 6)-α glycosidic bond formed by the C6 end of glucose in the glycoside chain (Satoh et al. 2003; Zeeman et al. 2010; Tanaka et al. 2004). Although SBE and SS are mainly involved in amylopectin synthesis, recent studies show that they also might be involved in amylose synthesis (Li et al. 2015; Yu et al. 2018). However, little is known about the details of how the individual isoforms of SBE and SS contribute to the amylose and amylopectin chain-length distribution (CLD) (Li et al. 2017; Li et al. 2015; Guan and Preiss 1993). One isoform of the starch debranching enzymes (DBEs) hydrolyzes widely-spaced (1 → 6)-α bonds of the polysaccharide chain to achieve spacing between amylopectin branch points such that crystallization between amylopectin chains can occur (Nakamura et al. 1997). AGPase catalyzes glucose-1-phosphate and ATP to form pyrophosphate and ADPG (ADP-glucose). ADPG is the initial glucose-based donor of starch biosynthesis and a substrate for starch synthesis, whose concentration directly affects the rate and efficiency of starch synthesis (Baroja-Fernandez et al. 2012; Bowsher et al. 2007).

The present study looks for the presence of concerted actions between enzymes, involved in the synthesis of both amylopectin and amylose by investigating 19 natural rice samples (Table 1). The range of amylose, up to 28%, goes to significantly higher values than normally encountered in rice. Enzyme actions are investigated through studying the chain-length distributions (CLDs: the number or weight distributions of monomer units in individual chains), these being measured by enzymatically debranching the starch and determining the molecular weight distributions of the resulting linear polymers by fluorophore-assisted carbohydrate electrophoresis (FACE) for amylopectin chains and size-exclusion chromatography (SEC, a type of gel-permeation chromatography, GPC) for amylose chains. One of the novel aspects of the present study is to parameterize these CLDs using biosynthesis-based models, which provide a very good fit to these data. The result is a comparatively small set of parameter values, representing various biosynthesis enzyme activities, which can then be used to find statistically valid correlations among themselves, thereby revealing interactions between SBE, DBE, SS, and GBSS.

**Table 1:** Rice samples from different botanical sources

Number	Name	Country of origin
1	IRTP 19771-G1	Ivory Coast
2	GERVEX 1686-C1	Greece
3	IRGC 64858–1	South Korea
4	Qingsiai 16B	China
5	IRGC 12872–1	New Zealand
6	UPR 191–66	India
7	IRGC 62683–1	China
8	MONOLAYA	United States
9	GERVEX 1638-C1	United States
10	K24	Uganda
11	NERICA-L^−1^	Africa
12	IRGC 70371–1	China
13	IRGC 28036–1	Pakistan
14	IRGC 46659–1	India
15	IRGC 53437–1	China
16	Yunlu 103	China
17	IRGC 38606–1	India
18	IRGC 3272–1	Argentina
19	Wuxiangjing 14	China

## Results and Discussion

### Fitting Parameters

**Fig. 1:**
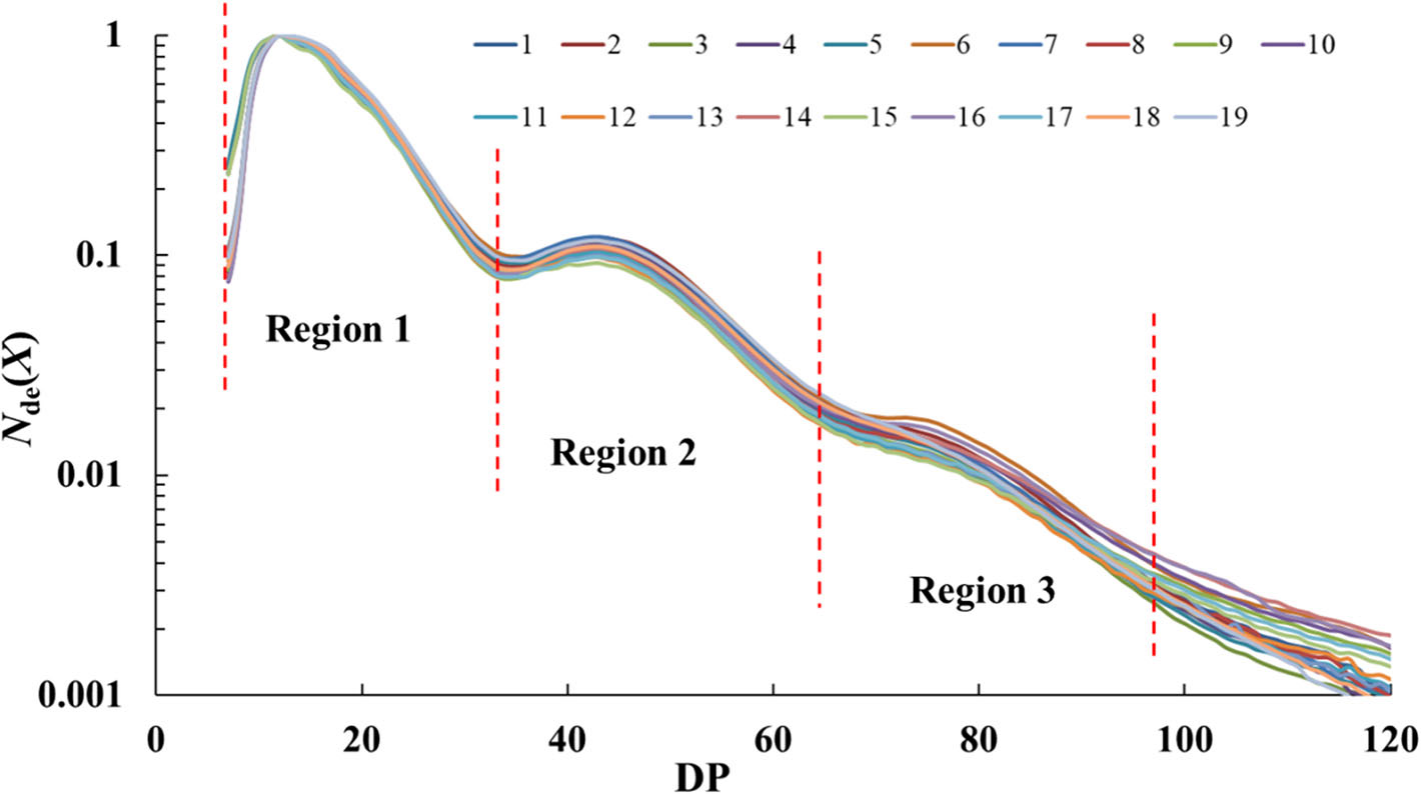
CLDs from FACE analysis, normalized to the highest amylopectin peak

The amylopectin CLDs are shown in Fig. 1, normalized to the highest peak. All 19 samples show typical amylopectin distributions, and three regions can be seen. The amylopectin model fitting parameters are shown in Table 2 with the fits shown in the Supporting Information (Additional file 1: Figure S1-a and S1-b). Amylopectin chains are grouped into A, B and C chain, and B chain can be further divided into B1, B2, B3 chains (Tester et al. 2004). In the amylopectin fitting model, region 1 can be generally considered representing A and B1 chains, region 2 represents B2 chains and region 3 represents B3 chains. As detailed in the methods sections, the biosynthesis parameters (which are the ratio of activities of branching and synthase enymes) β_(i)_ and β_(ii)_ are calculated based on CLDs of region1, β_(iii)_ and β_(iv)_ are calculated based on those of region 2, and β_(v)_ and β_(vi)_ on those of region 3. For the biosynthesis parameters representing the relative rates of chain elongation, *h*_(iii/i)_ is the ratio of region 2 height to region 1 height, while *h*_(v/i)_ is the ration of region 3 height to region 1 height.

**Table 2:** Parameter values from fitting the biosynthesis model to the amylopectin CLDs of the 19 rice samples

Sample	*β*_(i)_ / 10^−3^	*β*_(ii)_ / 10^− 3^	*β*_(iii)_ / 10^− 3^	*β*_(iv)_ / 10^− 3^	*β*_(v)_ / 10^− 3^	*β*_(vi)_ / 10^− 3^	*h*_(iii/i)_ / 10^− 3^	*h*_(v/i)_ / 10^− 3^
1	113.0 ± 0.1	28.2 ± 0.7	57.5 ± 0.3	14.7 ± 0.2	44.7 ± 0.1	13.5 ± 0.2	104.2 ± 0.3	7.4 ± 0.1
2	113.5 ± 2.1	33.0 ± 1.6	60.9 ± 1.8	12.9 ± 0.3	46.0 ± 4.7	10.0 ± 1.9	111.1 ± 5.3	7.7 ± 0.6
3	97.3 ± 0.7	29.8 ± 0.4	54.6 ± 0.1	14.1 ± 0.6	43.3 ± 1.0	10.8 ± 0.3	95.8 ± 2.0	5.8 ± 0.2
4	117.0 ± 1.3	26.7 ± 0.5	61.3 ± 2.2	13.6 ± 1.8	43.7 ± 1.1	12.6 ± 0.7	111.6 ± 2.6	7.7 ± 0.2
5	100.1 ± 1.3	33.4 ± 0.8	55.6 ± 0.3	16.2 ± 0	49.4 ± 0.6	15.5 ± 1.9	99.8 ± 3.2	6.7 ± 0.3
6	97.4 ± 0.2	27.0 ± 1.9	53.7 ± 0.1	14.4 ± 0.2	54.6 ± 0.5	8.5 ± 0.3	105.6 ± 1.6	10.5 ± 1.1
7	97.4 ± 6.5	37.3 ± 3.8	53.7 ± 2.4	18.3 ± 0.9	43.2 ± 4.2	21.1 ± 1.5	115.5 ± 2.8	7.8 ± 0.3
8	119.6 ± 0.8	23.2 ± 0.4	56.8 ± 0.7	17.4 ± 0.0	40.8 ± 0.8	13.5 ± 1.6	103.9 ± 1.6	8.1 ± 0.5
9	123.0 ± 0.6	21.4 ± 0.4	53.3 ± 0.7	21.4 ± 0.1	37.8 ± 4.5	12.7 ± 1.4	94.1 ± 0.2	7.1 ± 0.2
10	121.5 ± 0.2	26.1 ± 2.1	55.8 ± 0.2	17.9 ± 0.3	39.4 ± 1.5	6.9 ± 0.5	107.7 ± 1.9	8.0 ± 0.2
11	118.2 ± 2.4	25.0 ± 1.0	52.7 ± 2.9	21.0 ± 2.2	37.8 ± 3.1	15.4 ± 2.2	99.1 ± 1.9	6.8 ± 0.4
12	123.5 ± 0.9	19.2 ± 0.7	57.0 ± 4.3	13.4 ± 2.9	35.9 ± 1.5	17.6 ± 1.0	88.6 ± 0.2	6.0 ± 0.4
13	115.2 ± 3.9	32.7 ± 1.5	54.2 ± 0.6	17.2 ± 0.2	37.4 ± 0.7	13.7 ± 0.8	96.9 ± 5.5	6.9 ± 0.6
14	106.2 ± 0.7	25.8 ± 0.9	52.0 ± 0.7	18.0 ± 0.4	35.2 ± 1.2	7.1 ± 0.3	97.7 ± 5.5	7.3 ± 0.3
15	103.8 ± 0.1	27.9 ± 2.6	50.4 ± 3.1	18.3 ± 1.7	38.1 ± 5.2	11.6 ± 1.1	82.3 ± 2.0	6.1 ± 0.3
16	122.5 ± 0.5	15.9 ± 1.3	54.5 ± 0.8	15.0 ± 3.8	32.9 ± 4.9	17.3 ± 2.3	102.6 ± 1.6	9.3 ± 0.1
17	110.9 ± 0.2	32.7 ± 3.2	51.7 ± 4.3	18.4 ± 2.8	31.1 ± 0.2	16.8 ± 2.9	93.3 ± 2.7	6.6 ± 0.3
18	115.6 ± 1.6	35.1 ± 0.7	50.2 ± 0.3	19.3 ± 0.6	41.1 ± 3.8	9.5 ± 1.1	104.5 ± 2.1	6.6 ± 0.1
19	111.2 ± 1.7	30.3 ± 1.1	49.6 ± 1.3	18.2 ± 0.6	38.8 ± 2.1	9.8 ± 0.4	105.6 ± 4.1	6.1 ± 0.5

**Fig. 2:**
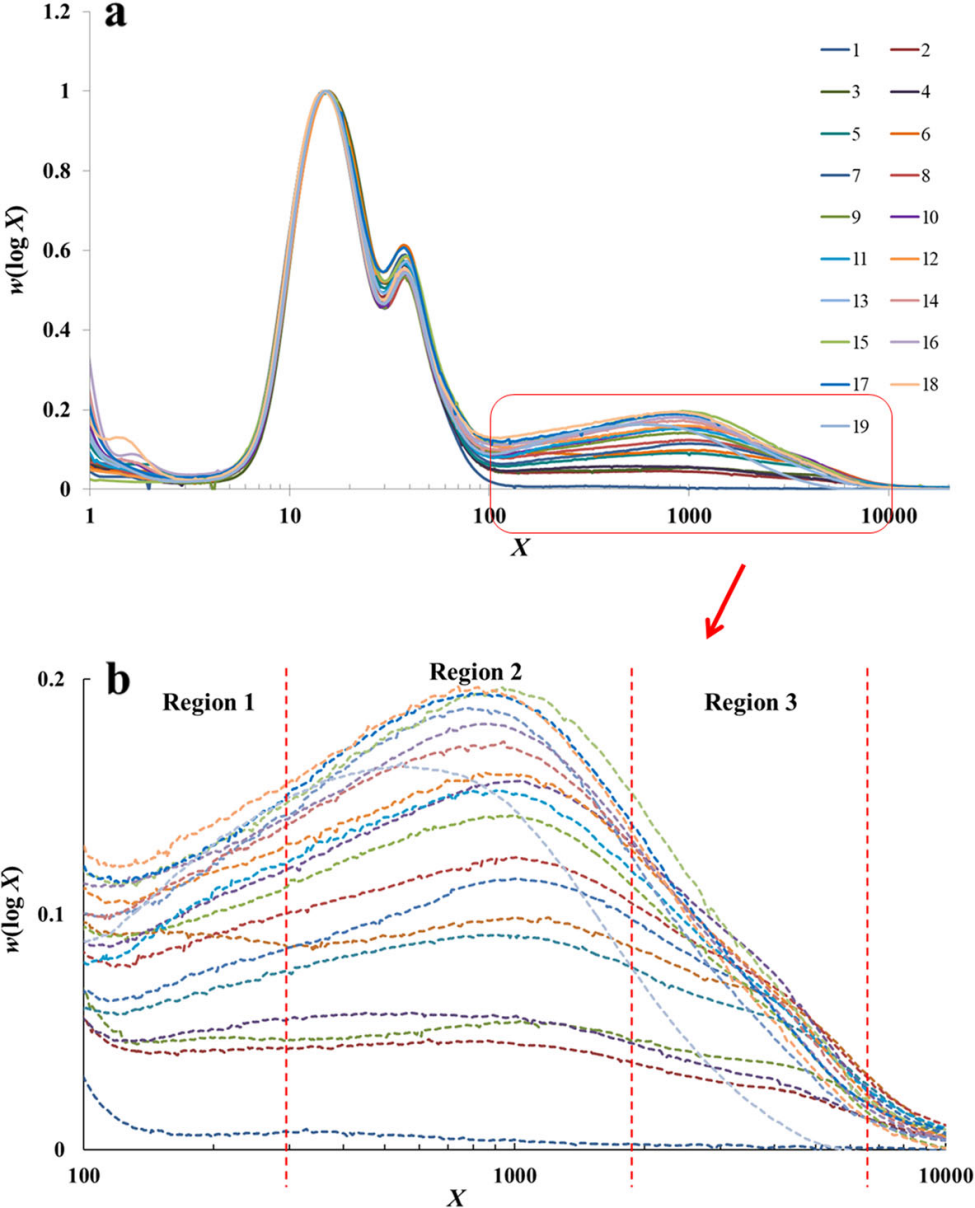
SEC weight CLDs of the whole range of debranched starch branches. All distributions are normalized to the highest amylopectin peak

The weight CLDs of amylopectin and amylose from SEC are given in Fig. 2, normalized to the highest peak (which is for short amylopectin chains). One normally divides CLDs into amylose and amylopectin portions where the CLD shows a minimum, which is almost always close to degree of polymerization (abbreviation DP, given the symbol *X*) close to 100. Those for ≲ 100 are amylopectin chains and those with *X* ≳100 are amylose chains. While SEC is needed for the amylose CLDs, the SEC amylopectin CLDs are not used here because FACE can give a much better resolution for DP ≲ 180. Amylose CLDs were fitted with the biosynthesis-based models described previously (Wu et al. 2013a; Yu et al. 2019; Nada et al. 2017). The final outcomes of these models is that various features in different regions of the CLDs can be expressed in terms of just two parameters: β (with appropriate subscripts for amylopectin or amylose and for which feature), which is the ratio of the activities of the starch branching (SBE) and starch synthase (SS) dominating the CLD in that feature, and *h* (again with appropriate subscripts) which is the relative activity of the dominant SS in that region (Additional file 1: Figure S1-c and S1-d). The amylose fitting parameters are shown in Table 3.

**Table 3:** Parameter values from fitting the biosynthesis model to the amylose CLDs

Sample	Amylose content / %	*β*_Am, (i)_ / 10^− 3^	*β*_Am,(ii)_ / 10^− 3^	*β*_Am,(iii)_ / 10^− 3^	*h*_Am, (i)_ / 10^− 3^	*h*_Am,(ii)_ / 10^− 3^	*h*_Am,(iii)_ / 10^− 3^
1	1.1 ± 0.7	N/A	N/A	N/A	N/A	N/A	N/A
2	9.6 ± 0.9	11.1 ± 0.0	2.6 ± 0.0	0.6 ± 0.0	20.4 ± 0.0	23.9 ± 0.2	21.8 ± 2.6
3	10.9 ± 0.2	10.7 ± 0.2	2.5 ± 0.0	0.6 ± 0.0	25.1 ± 1.1	34.0 ± 1.5	30.4 ± 2.2
4	11.9 ± 0.6	10.3 ± 0.5	2.7 ± 0.0	0.7 ± 0.0	28.3 ± 1.3	36.5 ± 1.3	26.4 ± 0.0
5	15.7 ± 0.6	10.7 ± 0.0	2.7 ± 0.0	0.7 ± 0.0	33.3 ± 0.1	56.9 ± 0.3	45.6 ± 2.3
6	17.1 ± 0.8	10.6 ± 0.1	2.7 ± 0.1	0.7 ± 0.0	36.7 ± 0.9	60.6 ± 2.9	56.2 ± 1.0
7	20.5 ± 0.6	11.0 ± 0.5	2.7 ± 0.0	0.8 ± 0.0	37.8 ± 1.0	68.1 ± 0.5	60.2 ± 1.4
8	22.2 ± 1.0	10.6 ± 0.4	2.7 ± 0.0	0.8 ± 0.0	46.8 ± 1.5	72.9 ± 3.2	65.7 ± 1.9
9	23.6 ± 0.2	10.5 ± 0.2	2.7 ± 0.0	0.8 ± 0.0	48.9 ± 1.9	85.4 ± 1.9	63.3 ± 1.7
10	24.0 ± 0.5	11.4 ± 0.0	2.9 ± 0.1	0.8 ± 0.0	46.3 ± 1.6	69.8 ± 4.3	77.6 ± 1.7
11	24.2 ± 1.4	10.3 ± 0.3	2.7 ± 0.0	0.8 ± 0.0	53.3 ± 1.9	90.1 ± 5.4	69.4 ± 1.2
12	25.1 ± 0.2	10.2 ± 0.1	2.7 ± 0.0	0.9 ± 0.0	57.3 ± 1.1	94.3 ± 0.0	67.6 ± 1.3
13	25.7 ± 0.7	11.1 ± 0.6	2.8 ± 0.0	0.9 ± 0.0	54.1 ± 1.2	112.5 ± 2.3	70.4 ± 1.9
14	26.3 ± 1.1	11.0 ± 0.8	2.8 ± 0.1	0.9 ± 0.0	57.7 ± 0.7	102.8 ± 5.9	76.0 ± 1.8
15	26.8 ± 0.9	11.6 ± 0.2	2.8 ± 0.0	0.9 ± 0.0	61.6 ± 0.6	113.9 ± 4.5	80.4 ± 1.5
16	26.9 ± 0.6	11.8 ± 0.7	2.9 ± 0.0	0.9 ± 0.0	60.6 ± 1.1	109.2 ± 1.3	76.9 ± 1.2
17	26.9 ± 0.5	11.6 ± 0.5	2.9 ± 0.1	1.0 ± 0.0	62.8 ± 0.7	111.5 ± 5.2	82.1 ± 1.6
18	28.1 ± 0.4	11.5 ± 0.5	2.9 ± 0.0	1.0 ± 0.0	72.7 ± 1.5	115.2 ± 5.6	85.4 ± 1.8
19	23.7 ± 1.2	10.6 ± 0.0	2.8 ± 0.0	0.8 ± 0.0	51.2 ± 0.3	86.1 ± 1.0	67.5 ± 1.2

### Correlations between Parameters for Amylopectin and Amylose

It is noted that the complete chain-length distribution can be used to calculate the degree of branching through the formula (Wu et al. 2013b): $\mathrm{DB}=\frac{\sum_X{N}_{de}(X)}{\sum_XX{N}_{de}(X)}$, which is an exact result arising from the definition of the degree of branching and so this quantity is not an independent structural variable, given a complete parameterization of the CLD, as done here. However, it is a useful and commonly used property, so correlations with the independent structural are listed here. The correlations between amylopectin and amylose structural parameters are listed in Table 4. It is seen that β_Am,(iii)_ significantly negatively correlates with β_(iii)_, and strongly negatively correlates with β_(v)_. β_Am,(iii)_ represents long amylose chains, and β_(iii)_ and β_(v)_ represent intermediate and long amylopectin chains. This suggests that the synthesis of long amylose chains competes with the syntheses of intermediate and long amylopectin chains, especially the long amylopectin chains, and perhaps these two syntheses could happen at the same site in starch granules. It has been shown that GBSSI is the dominant enzyme controlling long amylose chain synthesis (Li et al. 2015; Wang et al. 2015). SSIII is believed to be the main SS and SBEI the main SBE involved in the synthesis of longer amylopectin chains (DP > 25) (Bowsher et al. 2007; Zhang et al. 2008). It is seen that *h*_(iii/i)_ and *h*_(v/i)_ have no significant correlation with *h*_Am,(iii)_, which indicates that the interaction between SSIII and GBSSI is not significant. SBEI can transfer long glucosyl chains (DP > 20) to form long amylopectin branches, while GBSSI can elongate amylose chains with long glucosyl chains (DP > 20) (Denyer et al. 2001; Wang et al. 2015). The correlation results suggest that both GBSSI and SBEI compete for the similar substrates (long glucosyl chains) during starch biosynthesis, an inference not previously reported. The correlation result also shows that β_Am,(iii)_ is not significantly correlates with β_(i)_ and β_(ii)_, which shows that GBSSI has little interaction with SBEIIb in rice. Future experiments are needed to analyze the activity of different enzyme isoforms (especially GBSSI and SBEI) and the interactions between different isoforms.

**Table 4:** Pearson correlation coefficients between amylopectin and amylose structural parameters ^1^. AC = amylose content

correlation coefficients	AC	β_Am, (i)_	β_Am,(ii)_	β_Am,(iii)_	*h*_Am, (i)_	*h*_Am,(ii)_	*h*_Am,(iii)_	β_(i)_	β_(ii)_	β_(iii)_	β_(iv)_	β_(v)_	β_(vi_)	*h*_(iii/i)_	*h*_(v/i)_
AC	1														
β_Am, (i)_	0.369	1													
β_Am,(ii)_	0.750^**^	0.597^**^	1												
β_Am,(iii)_	0.937^**^	0.483^*^	0.829^**^	1											
*h*_Am, (i)_	0.967^**^	0.407	0.775^**^	0.960^**^	1										
*h*_Am,(ii)_	0.963^**^	0.417	0.721^**^	0.938^**^	0.965^**^	1									
*h*_Am,(iii)_	0.977^**^	0.438	0.772^**^	0.889^**^	0.946^**^	0.925^**^	1								
β_(i)_	0.380	−0.061	0.406	0.472	0.386	0.285	0.272	1							
β_(ii)_	−0.246	0.204	−0.046	− 0.197	−0.222	− 0.179	−0.181	− 0.594^*^	1						
β_(iii)_	−0.685^**^	− 0.318	−0.376	− 0.586^*^	−0.682^**^	− 0.711^**^	− 0.731^**^	0.244	−0.162	1					
β_(iv)_	0.633^**^	0.104	0.348	0.538^*^	0.560^*^	0.561^*^	0.636^**^	0.147	0.100	− 0.683^**^	1				
β_(v)_	−0.712^**^	− 0.321	−0.550^*^	− 0.798^**^	− 0.697^**^	− 0.697^**^	− 0.615^**^	− 0.549^*^	0.357	0.324	−0.383	1			
β_(vi)_	0.123	−0.079	−0.094	0.120	0.061	0.159	0.049	0.052	−0.033	0.087	0.018	−0.252	1		
*h*_(iii/i)_	−0.433	−0.061	− 0.129	−0.400	− 0.477^*^	−0.534^*^	− 0.391	−0.053	0.313	0.398	−0.192	0.451	−0.063	1	
*h*_(v/i)_	−0.139	−0.120	0.008	−0.217	− 0.202	−0.196	− 0.064	0.002	− 0.260	0.283	− 0.243	0.385	− 0.090	0.521^*^	1

^1*^. Correlation is significant at the 0.05 level

^**^. Correlation is significant at the 0.01 level

Interestingly, there is no correlation observed between β_Am, (i)_ and β_Am,(ii)_ and amylopectin structural parameters. Earlier studies have shown that, besides GBSSI, some branching enzymes and soluble starch synthases are also involved in short amylose chain biosynthesis (Buleon et al. 1998; Li et al. 2015). This might mean that the synthesis of short amylose chains involves more enzymes than those for long amylopectin chains, which could explain why the structural parameters of short amylose chains showed no significant correlation with those of amylopectin.

It is seen that β_(iii)_ and β_(v)_ show negative correlation with *h*_Am,(i,)_ *h*_Am,(ii)_ and *h*_Am,(iii)_. The amylose *h* parameters are of course strongly positively correlated with amylose content (Table 4). GBSSI is believed to play a key role in determining the amylose content of cereal grains, and it competes with SBEI during starch biosynthesis (as discussed above). Thus, the parameters in each amylose region are negatively correlated with β_(iii)_ and β_(v)_ values. These results might indicate that some elongation of amylose chains happens inside of starch granules, where the synthesis of amylose and amylopectin can compete for substrates and enzymes.

It is found that β_(iv)_ is positively correlated with β_Am,(iii)_, *h*_Am, (i)_, *h*_Am,(ii)_ and *h*_Am,(iii)_, the opposite of what is seen with β_(iii)_ and β_(v)_. β_(iv)_ is negatively correlated with β_(iii)_, which is an expected result because the amylopectin model assumes (Wu et al. 2013a) that for rice, the branching enzymes of in these region compete for substrate. Hence this positive correlation is probably a statistical coincidence.

It is seen that *h*_(iii/i)_ has a weak negative correlation with *h*_Am, (i)_ and *h*_Am,(ii)_. If this weak correlation is indeed the case, this might be because same SBE isoforms participate in the synthesis of both intermediate amylopectin and short amylose chains. It also could be a statistical coincidence, because it is only a weak correlation and *h*_(iii/i)_ does not have a significant correlation with *h*_Am,(iii)_, while the latter is strongly correlated with *h*_Am, (i)_ and *h*_Am,(ii)_. The internal correlations between amylopectin structural parameters, and internal correlations between amylose structural parameters, are not discussed here as they are not germane to the aim of the present study, which is to look at which enzymes are involved in the synthesis of both amylopectin and amylose.

## Conclusions

Quantitative analysis of the structural parameters for amylopectin and amylose chain length distributions of nineteen natural rice samples indicate that GBSSI competes with SBEI for substrates during starch synthesis in rice. This might be of use for developing rice varieties with desirable amylose contents and CLDs. The results also confirm that GBSSI, SBE and SS are all involved in biosynthesis of short amylose chains.

## Methods

### Materials

Nineteen natural rice varieties were harvested from the same large field in Sanya, Hainan Province, China; details are listed in Table 1. Dimethyl sulfoxide (DMSO, GR grade for analysis) was purchased from Merck & Co., Inc. (Kilsyth, VIC, Australia). Protease from Streptomyces griseus (type XIV) and LiBr (ReagentPlus) were purchased from Sigma-Aldrich Pty. Ltd. (Castle Hill, NSW, Australia). Isoamylase (from Pseudomonas sp.) was purchased from Megazyme International, Ltd. (Wicklow, Ireland). 8-Aminopyrene-1,3,6-trisulfonate (APTS), included in the *Carbohydrate Labelling and Analysis Kit*, was purchased from BeckmanCoulter (Brea, USA). Other chemical reagents were analytical grade and used as received.

### Processing of Grains

Rice grains (5 g) were precooled in liquid nitrogen for 5 min before being ground to rice flour using a cryo-grinder (MM400, Netsch, Germany). Grinding at a low temperature reduces the damage of the molecular and granular structure of starch. All samples were ground for 2 min at 30 s^− 1^ following the method of (Hasjim et al. 2012).

### Preparation of Debranched Samples

Rice starch was extracted following method of (Syahariza et al. 2010) with some modifications. Rice flour (7–9 mg) was treated by protease in tricine buffer (0.5 mL, 2.5 Units/mL) in a thermomixer (Thermomixer Comfort, Hanbury, Germany) at 37 °C for 60 min, then treated with sodium bisulfite solution (0.5 mL, 0.45% w/w) at 37 °C for 30 min. The solution was centrifuged at 4000 rpm for 10 min, and the supernatant containing most of protein was removed. The precipitate was dissolved in DMSO/LiBr solution (1.5 mL, 0.5% w/w) in a thermomixer at 80 °C for 24 h, and inverted occasionally by hand to ensure a homogenous mixture. Then the mixture was centrifuged at 4000 rpm for 10 min, and the precipitate (mostly proteins and non-starch polysaccharides) was discarded. Starch was precipitated from the supernatant by adding 5 times the volume of absolute ethanol. The extracted starch was dispersed in boiling deionized water (0.9 mL) with occasional gentle shaking for at least 1 h. Then it was mixed with sodium azide solution (5 μL, 0.04 g mL^− 1^), acetate buffer solution (0.1 mL, 0.1 M, pH 3.5) and isoamylase (2.5 μL, 1000 U/mL), and incubated at 37 °C for 3 h. The debranching process was stopped by adding NaOH solution (0.1 M) to adjust the pH to 7, and the solution was incubated at 80 °C for 1 h. All the samples were freeze-dried overnight and stored in a desiccator for SEC and FACE analysis.

### SEC Analysis and Amylose Content

The debranched starch (~ 4 mg) was dissolved in 1 mL DMSO/LiBr (0.5%, w/w) at 80 °C overnight, and the solution was transferred into SEC vials for SEC analysis. The SEC separation was carried out with a Shimazu LC20 system (Kyoto, Japan) with a combination of Gram pre-column, Gram 1000 column and Gram 100 column (Polymer Standards Service, Mainz, Germany) in sequence. The eluent used in this study was DMSO-LiBr solution (0.5%, w/w), and the flow rate was set at 0.6 mL/min. The column temperature was set at 80 °C and the differential refractive index (DRI) detector temperature was set at 45 °C. The DRI signal was calibrated with a series of pullulan standards ranging from 342 to 2.35 × 10^6^ Da, and these standards provided universal calibration curves to relate elution volume *V*_el_ with hydrodynamic volume *V*_h_. For a linear polymer such as debranched starch, its *V*_h_ can in turn be converted to the degree of polymerization (DP – the number of monomer units in a chain) *X* using the Mark–Houwink equation: ${V}_{\mathrm{h}}=\frac{2}{5}\;\frac{K{\left(X\;{M}_0+18\right)}^{1+\alpha }}{N_{\mathrm{A}}}:{M}_0=162.2$ is the molecular weight of the anhydroglucose monomer unit and 18 is that of the additional water in the end groups, *N*_A_ is Avogadro’s constant, K and α for linear starch chains in the eluent of DMSO/LiBr at 80 °C are 1.5 × 10^− 4^ dL g–1 and 0.743, respectively (Vilaplana and Gilbert 2010a; Liu et al. 2010). The result is the SEC weight distribution *w* (log*X*). Although of course SEC signals vary with elution times in different runs, such variation is explicitly taken into account by the SEC calibration used here; that is why one must never present SEC data as a function of elution volumes (Gidley et al. 2010), which are instrumental quantities, but instead be presented as functions of molecular properties such as *X* and *R*_h_.

The amylose content was found by calculating the ratio of the area under the curve (AUC) of amylose composition (at the value of *X* where there is a clear division between short amylopectin chains and long amylose chains, at *X* ~ 100) to the AUC of the entire starch composition (Vilaplana et al. 2012).

The value of the amylose content depends on the method of measurement (Gray-Weale and Gilbert 2009), and is often therefore denoted “apparent amylose content”. Separating the amylose and amylopectin chains at the DP (~ 100) where there is a minimum, as we do here, is a simplification, as it ignores contributions to amylopectin chains by very short amylose ones, and to amylose chains by very long amylopectin ones. All 19 rice samples we used in this experiment have moderate amylose content (the highest is 28.1%), and they all show normal debranched starch distributions. While there can be some extra-long amylopectin chains, starch with a significant number of such chains is high-amylose starch (e.g. (Li et al. 2019)), and their debranched distributions are very different from normal starch. Thus the samples used in this study unlikely have very long amylopectin chains, and the amylose content calculated from the overall CLD is close to true amylose content. For high-amylose starches, the whole question of defining amylose content becomes much more complex, and can really only be performed using two-dimensional analysis (Vilaplana and Gilbert 2010b; Vilaplana et al. 2012; Vilaplana et al. 2014), where one dimension is the length of individual chains, and the other is the total molecular size.

### Amylopectin CLDs

The CLDs of the amylopectin component were analyzed using FACE. Debranched starch (0.2 mg) was labeled with 1.5 μL APTS solution (5 mg of APTS in 50 μL of 15% glacial acetic acid) and 1.5 μL sodium cynoborohydride, and incubated in a thermomixer at 60 °C for 4 h in the dark. Then 80 μL deionized water was added to the sample, and the solution was vortexed at a low speed to until dissolution was complete. 50 μL of the solution was transferred to FACE micro-vials for analysis, using a P/ACE MDQ plus system (Ab Sciex, US). The analysis was conducted following the method of (Bai et al. 2017). FACE directly gives the number CLD, *N*_de_(*X*), the number distribution of chains containing *X* monomer units after debranching. The relation between the number and weight distributions is *w* (log*X*) = *X*^2^ *N*_de_(*X*) (Castro et al. 2005).

The *N*_de_(*X*) were fitted with the Wu-Gilbert model (Wu and Gilbert 2010; Wu et al. 2013a). The model assumes that the CLD is controlled by isoforms of the three types of starch biosynthetic enzymes, SS, SBE and DBE. It also assumes that different “enzyme sets”, comprising one isoform of each type, contribute to the overall CLD, with different sets being major, but not exclusive, sources of chains for different ranges of DP. The rather complex equations quantifying the CLD are expressed in terms of two parameters for each set: β_*i*_ and *h*_*i*_, being respectively the ratio of the activity of SBE to that of SS in set *i*, and the relative contribution of that set to the overall CLD; in some regions, two sets can compete for the same substrates. For reasons discussed elsewhere (Wu et al. 2013a), the fit to the accurate CLD from FACE involves the parameters β_(i)_, β_(ii)_, β_(iii)_, β_(iv)_, β_(v)_, β_(vi)_, *h*_(iii/i)_ and *h*_(v/i)_. The fit uses publicly available code (Wu et al. 2013a).

### Amylose CLDs

The CLDs of amylose were fitted to a different mathematical model with similar assumptions, but this time implicitly involving GBSS as well (Nada et al. 2017; Yu et al. 2019). The treatment takes partial account of SEC band broadening. The amylose parameters for the *i*^th^ set are denoted β_Am,*i*_ and *h*_Am,*i*._. In this study, three different features in the amylose CLD can be distinguished, giving 6 parameters for the fitting: β_Am, (i)_, β_Am,(ii)_, β_Am,(iii)_, *h*_Am, (i)_, *h*_Am,(ii)_ and *h*_Am,(iii)_. Again the fitting was performed with publicly available code (Nada et al. 2017).

### Statistical Analysis

The correlation of amylopectin and amylose molecular structural parameters were analyzed with Pearson correlation analyses using IBM SPSS, to reveal the possible connections between amylose biosynthesis and amylopectin biosynthesis. Statistical significance was set at a probability level of 0.05.

## Supplementary information

**Additional file 1: Figure S1-a.** Experiment (“exp”, from FACE) and model-fitted (“cal”) number chain-length distributions (*N*_de_(*X*), arbitrary normalization) of debranched amylopectin (DP < 100) from sample 1 to 9. **Figure S1-b.** Experiment (“exp”, from FACE) and model-fitted (“cal”) number chain-length distributions (*N*_de_(*X*), arbitrary normalization) of debranched amylopectin (DP < 100) from samples 10 to 19. **Figure S2-a.** Experiment (“exp”, from SEC) and model-fitted (“fit”) weight chain-length distributions (*w* (log*X*), arbitrary normalization) of debranched amylose (DP > 100) from samples 2 to 10. **Figure S2-b.** Experiment (“exp”, from SEC) and model-fitted (“fit”) weight chain-length distributions (*w* (log*X*), arbitrary normalization) of debranched amylose (DP > 100) from samples 11 to 19.

## Data Availability

All data generated or analysed during this study are included in this published article and its supplementary information files.

## Abbreviations

APTS: 8-Aminopyrene-1,3,6-trisulfonic acid, trisodium salt
CLD: Chain length distribution
DBE: debranching enzyme
DP: Degree of polymerization
FACE: Fluorophore-assisted carbohydrate electrophoresis
GBSS: Granule-bound starch synthase
SBE: Starch branching enzyme
SEC: Size exclusion chromatography
SS: Starch synthase
