# Supplementary material for: Competition between Granule Bound Starch Synthase and Starch Branching Enzyme in Starch Biosynthesis
Source: Rice (N Y). 2019 Dec 23;12:96. doi: 10.1186/s12284-019-0353-3 (PMC6928174; doi:10.1186/s12284-019-0353-3)
Supplement: Supplementary file 1 — Additional file 1: Figure S1-a. Experiment (“exp”, from FACE) and model-fitted (“cal”) number chain-length distributions (Nde(X), arbitrary normalization) of debranched amylopectin (DP < 100) from sample 1 to 9. Figure S1-b. Experiment (“exp”, from FACE) and model-fitted (“cal”) number chain-length distributions (Nde(X), arbitrary normalization) of debranched amylopectin (DP < 100) from samples 10 to 19. Figure S2-a. Experiment (“exp”, from SEC) and model-fitted (“fit”) weight chain-length distributions (w (logX), arbitrary normalization) of debranched amylose (DP > 100) from samples 2 to 10. Figure S2-b. Experiment (“exp”, from SEC) and model-fitted (“fit”) weight chain-length distributions (w (logX), arbitrary normalization) of debranched amylose (DP > 100) from samples 11 to 19. [file 12284_2019_353_MOESM1_ESM.docx]

Supporting Information for

**Competition between granule bound starch synthase and starch branching enzyme in starch biosynthesis**

Huaxin Han^1,2^, Chuantian Yang^1,2^, Jihui Zhu^1,2^, Lixia Zhang^3^, Yeming Bai4, Enpeng Li^1,2^* and Robert G. Gilbert^1,2,4^*

^1^ Jiangsu Key Laboratory of Crop Genetics and Physiology, Key Laboratory of Plant Functional Genomics of the Ministry of Education, Jiangsu Key Laboratory of Crop Genetics and Physiology, College of Agriculture, Yangzhou University, Yangzhou 225009, P.R. China

^2^ Co-Innovation Center for Modern Production Technology of Grain Crops, Yangzhou University, Yangzhou 225009, China

^3^ Crop Breeding and Cultivation Research Institute, Shanghai Academy of Agricultural Sciences, Shanghai 201403,China

^4^ Centre for Nutrition & Food Sciences, Queensland Alliance for Agriculture & Food Innovations, The University of Queensland, Brisbane, QLD 4072, Australia

* Corresponding authors：

Enpeng Li, Email: lep@yzu.edu.cn, Phone: + 86 18252713020 &

Robert G. Gilbert, Email: [b.gilbert@uq.edu.au](mailto:b.gilbert@uq.edu.au). Phone: +61 7 3365 4809. Fax: +61 7 3365 118

**Figure caption**

**Figure S1-a.** Experiment (“exp”, from FACE) and model-fitted (“cal”) number train-length distributions (*N*de(*X*), arbitrary normalization) of debranched amylopectin (DP < 100) from samples 1 to 9.

**Figure S1-b.** Experiment (“exp”, from FACE) and model-fitted (“cal”) number train-length distributions (*N*de(*X*), arbitrary normalization) of debranched amylopectin (DP < 100) from samples 10 to 19.

**Figure S2-a.** Experiment (“exp”, from SEC) and model-fitted (“fit”) number train-length distributions (*N*de(*X*), arbitrary normalization) of debranched amylose (DP > 100) from samples 2 to 10.

**Figure S2-b.** Experiment (“exp”, from SEC) and model-fitted (“fit”) number train-length distributions (*w*(log*X*), arbitrary normalization) of debranched amylose (DP > 100) from samples 11 to 19.

**
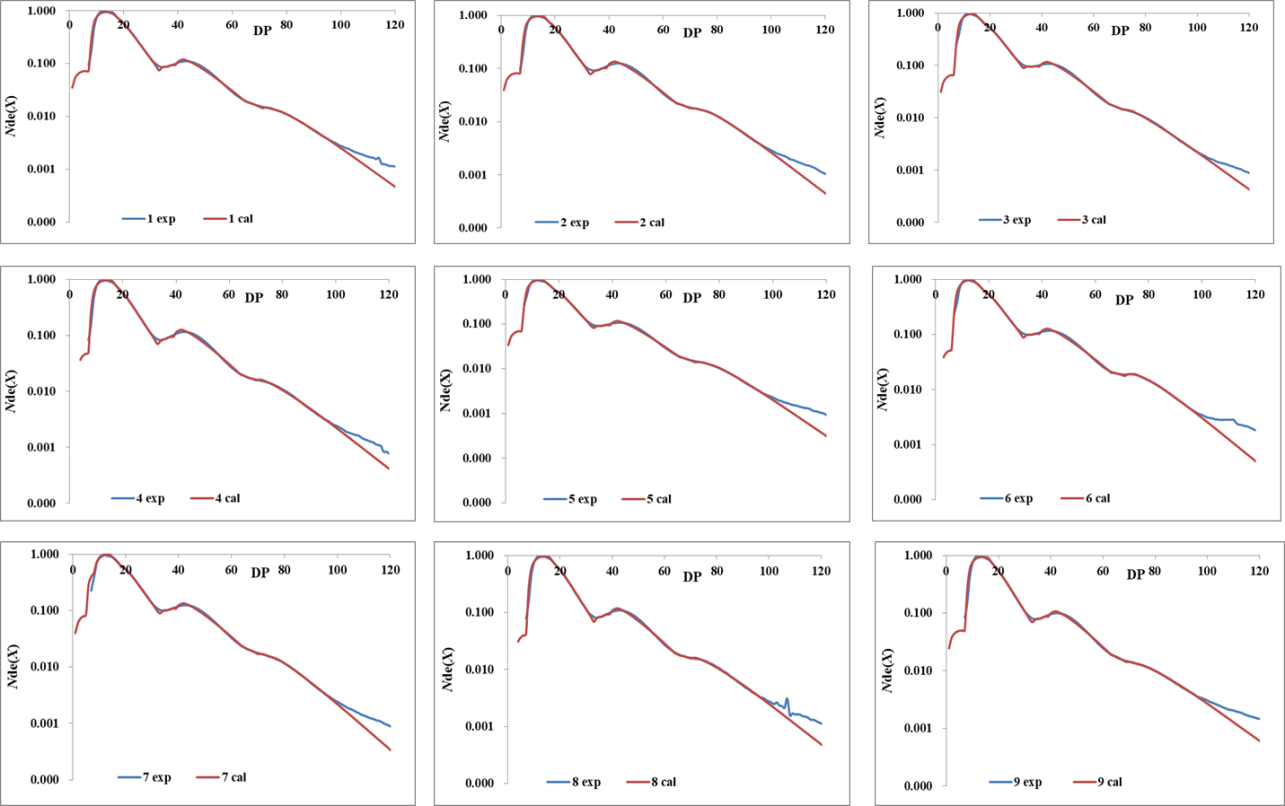
**

**Figure S1-a**

**
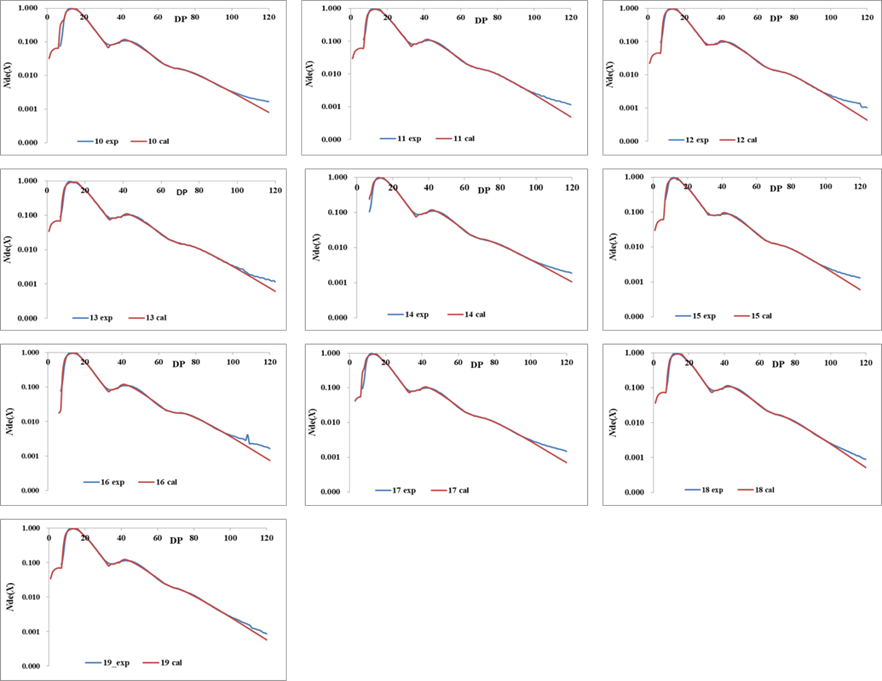
**

**Figure S1-b**

**
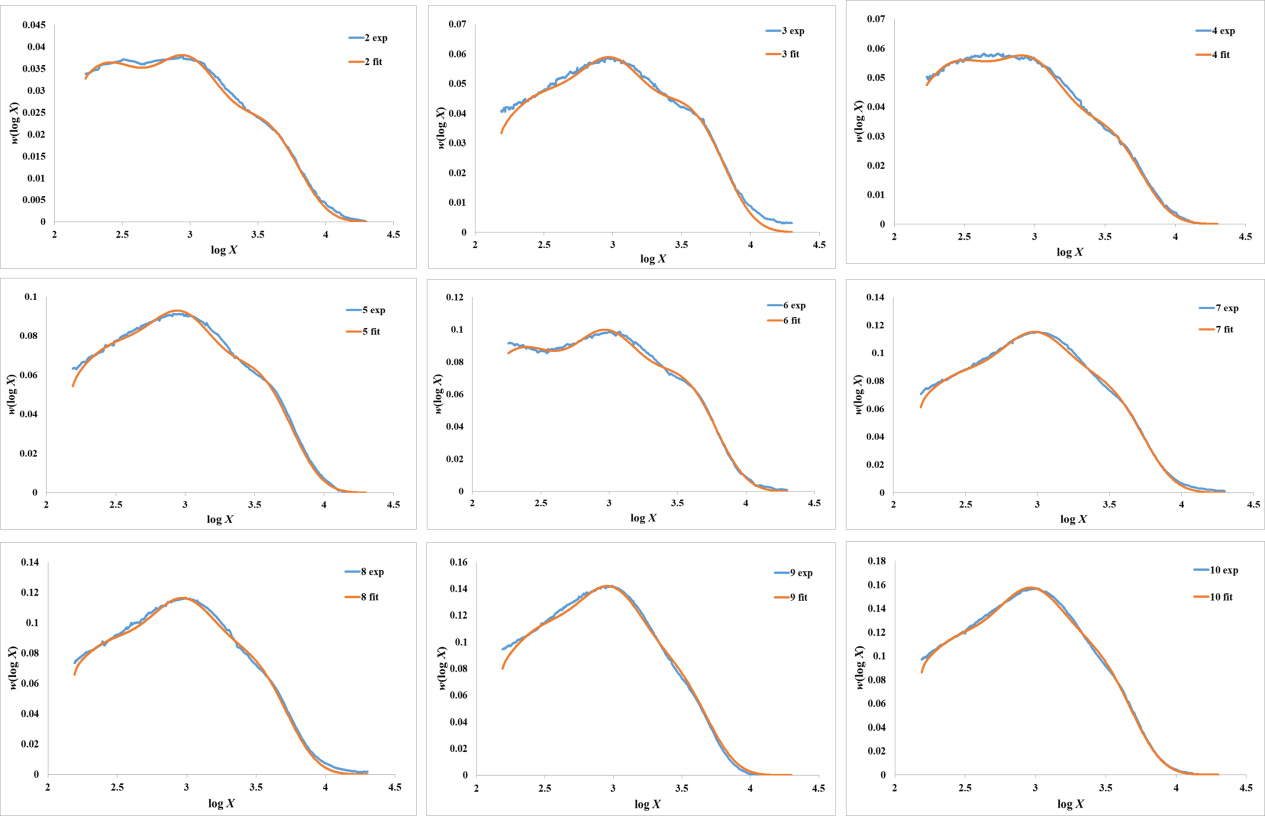
**

**Figure S2-a**

**
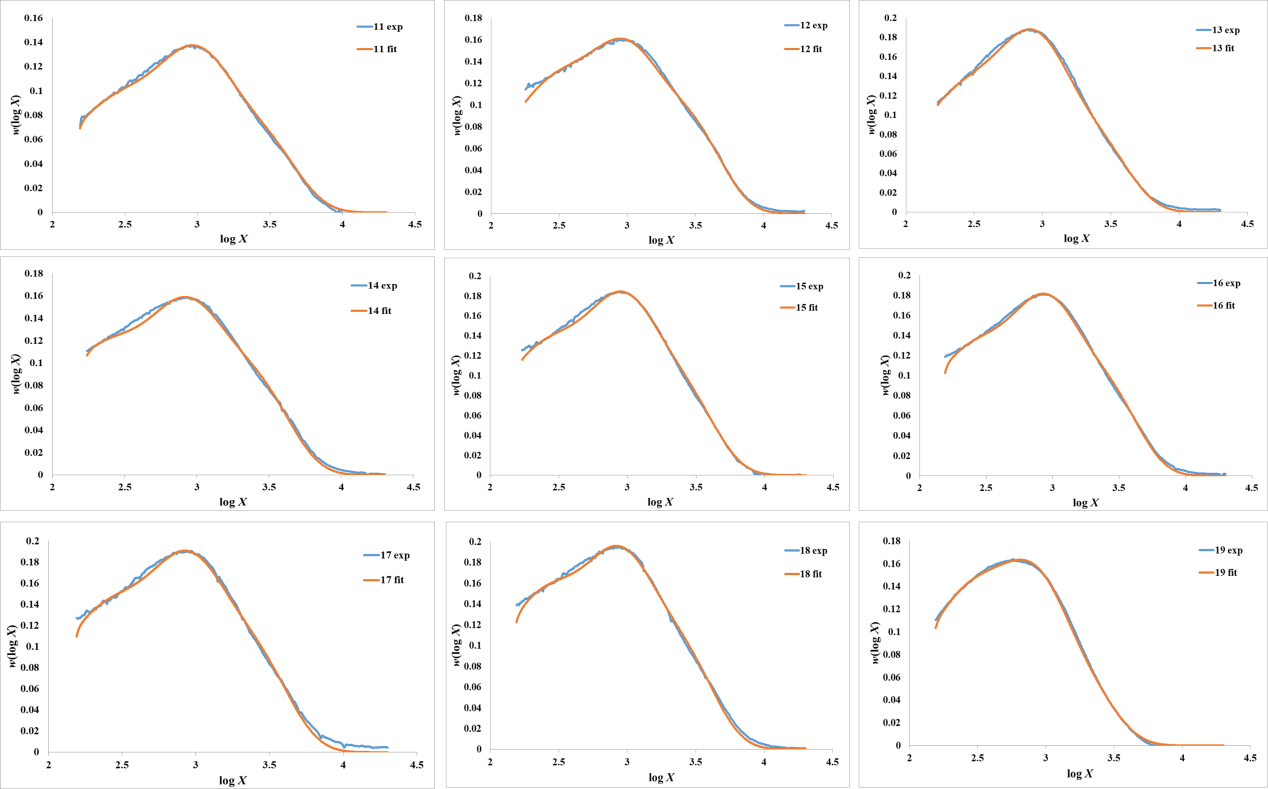
**

**Figure S2-b**
